# Betulinic acid accelerates diabetic wound healing by modulating hyperglycemia-induced oxidative stress, inflammation and glucose intolerance

**DOI:** 10.1093/burnst/tkac007

**Published:** 2022-04-09

**Authors:** Weiguo Xie, Weigang Hu, Zhuo Huang, Min Li, Hongyu Zhang, Xiaodong Huang, Paul Yao

**Affiliations:** 1 Institute of Burns, Tongren Hospital of Wuhan University (Wuhan Third Hospital), Wuhan, 430060 P.R. China; 2 Department of Hematology, Peking University Shenzhen Hospital, Shenzhen, 518036, P.R. China

**Keywords:** Betulinic acid, Diabetes, Inflammation, Oxidative stress, Wound healing, Nuclear factor κB, Glucose, Hyperglycemia

## Abstract

**Background:**

Diabetes significantly delays wound healing through oxidative stress, inflammation and impaired re-epithelialization that lead to defective regulation of the healing process, although the related mechanism remains unclear. Here, we aim to investigate the potential role and mechanism for the beneficial effect of betulinic acid (BA) on diabetic wound healing.

**Methods:**

The molecular effect of BA on hyperglycemia-mediated gene expression, oxidative stress, inflammation and glucose uptake was evaluated in endothelial, fibroblast and muscle cells. Burn injury was introduced to streptozotocin-induced diabetic rats and BA administration through either an intraperitoneal (IP) or topical (TOP) technique was used for wound treatment. Glucose tolerance was evaluated in both muscle tissue and fibroblasts, while oxidative stress and inflammation were determined in both the circulatory system and in wound tissues. The effect of BA on the wound healing process was also evaluated.

**Results:**

BA treatment reversed hyperglycemia-induced glucose transporter type 4 (GLUT4) suppression in both muscle and fibroblast cells. This treatment also partly reversed hyperglycemia-mediated suppression of endothelial nitric oxide synthase (eNOS), nuclear factor erythroid 2-related factor 2 (Nrf2) signaling and nuclear factor NFκB p65 subunit (NFκB p65) activation in endothelial cells. An *in vivo* rat study showed that BA administration ameliorated diabetes-mediated glucose intolerance and partly attenuated diabetes-mediated oxidative stress and inflammation in both the circulatory system and wound tissues. BA administration by both IP and TOP techniques significantly accelerated diabetic wound healing, while BA administration by either IP or TOP methods alone had a significantly lower effect.

**Conclusions:**

BA treatment ameliorates hyperglycemia-mediated glucose intolerance, endothelial dysfunction, oxidative stress and inflammation. Administration of BA by both IP and TOP techniques was found to significantly accelerate diabetic wound healing, indicating that BA could be a potential therapeutic candidate for diabetic wound healing.

Highlights

- Betulinic acid ameliorates hyperglycemia-mediated glucose transporter type 4 suppression and glucose intolerance in muscle cells.
- Betulinic acid attenuates hyperglycemia-mediated oxidative stress and inflammation and vascular dysfunction in endothelial cells.
- Betulinic acid administration by both intraperitoneal and topical methods significantly accelerates diabetic wound healing.

## Background

Wound healing is a complicated pathophysiological process that involves the steps of hemostasis, inflammation, proliferation, re-epithelialization and remodeling [1] as well as different cell types, including endothelial cells, fibroblasts, blood cells, muscle and stem cells [2–4]. Diabetes significantly delays wound healing, leading to several defects in the regulation of the normal healing process via consistent oxidative stress [5–7], inflammation [8] and impaired re-epithelialization [9,10]. Diabetes is associated with impaired wound healing [11] among other complications, resulting in a heavy social burden, severe health issues and high rates of morbidity and mortality [12,13]. Development of a potential mechanism and therapeutic treatment for diabetic wound healing is still urgently needed [14–16].

Betulinic acid (BA) is a pentacyclic triterpene product that is purified from natural products including pulsatilla chinensis [17]. It possesses many biological properties and impressive anti-tumor effects [18–20], although the related mechanism is still unclear [21]. Furthermore, it has been reported that BA induces endothelial nitric oxide synthase (eNOS) expression with potential vascular protective effects [22], attenuates oxidative stress by activation of the nuclear factor erythroid 2-related factor 2 (Nrf2) signaling pathway [23–25] with subsequent activation of antioxidant genes, such as superoxide dismutase 2 and glutathione peroxidase [23–27], and ameliorates inflammation by inhibition of the nuclear factor κB (NFκB) signaling pathway [28–30]. Interestingly, BA induces the expression of glucose transporter type 4 (GLUT4) [31] with potential anti-diabetic effects [32–35]. Based on the above observations, BA may appear to have a protective effect on diabetes-mediated oxidative stress, inflammation, vascular dysfunction and glucose intolerance, even as the potential mechanism remains largely unclear [36,37]. Thus, we hypothesize that BA may be a candidate for promoting diabetic wound healing [38].

In addition to the significant negative effects of diabetes complications on diabetic wound healing, diabetes also contributes to severe wound infection, which is usually the major reason for amputations in diabetic foot ulcers [39]. *Staphylococcus aureus* (*S. aureus*) has been identified as the dominant pathogen in diabetic infections such as diabetic foot ulcers [40]. Recent literature has shown that BA may be a potential candidate as a therapeutic agent for *S. aureus*-mediated infection [41]; additionally, it has been reported that BA has promising antimicrobial [42,43] and anti-viral effects [17,44]. These reports indicate that BA may be a promising candidate for diabetic wound healing due to its beneficial effects on diabetes-mediated infection and vascular damage, in addition to its anti-diabetic effects [32–35].

In this study, we aimed to investigate the effect of BA on diabetic wound healing. The results of our *in vitro* study showed that BA partly reversed hyperglycemia-mediated GLUT4 suppression in muscle cells and fibroblasts. A similar effect was observed in partly reversing hyperglycemia-mediated eNOS suppression, NFκB signaling activation and Nrf2 suppression in endothelial cells. An *in vivo* study in rats showed that BA administration partly reversed hyperglycemia-mediated oxidative stress and inflammation in the circulatory system and wound tissues. Similar effects were seen on diabetes-mediated glucose intolerance in muscles and fibroblasts. Furthermore, BA administration by both intraperitoneal (IP) and topical (TOP) treatment significantly accelerated cutaneous burn injury healing in diabetic rats. We conclude that BA accelerates diabetic burn injury healing by modulating hyperglycemia-induced oxidative stress, inflammation and glucose intolerance.

## Methods

An expanded Materials and Method section is provided in Supplementary Information (see online supplementary material, Data S1) and the primers used in this study are shown in Table S1 (see online supplementary material).

### Materials and reagents

Human primary aorta smooth muscle cells (HASMCs, #PCS-100-012) were purchased from ATCC and maintained in vascular cell basal medium supplemented with vascular smooth muscle growth kit. Antibodies for eNOS (sc-376751), GLUT4 (sc-53566) and NFκB p65 (sc-8008) were obtained from Santa Cruz Biotechnology (Shanghai, China). The transcriptional activity of NFκB p65 was determined by the NFκB p65 transcription factor assay kit (#ab133112 from Abcam) according to the manufacturer’s instructions and BA (#B8936) was purchased from Sigma (Shanghai, China).

### DNA methylation analysis

Human genomic DNA was extracted and purified from HASMCs, treated by bisulfite modification and then amplified by the following primers: methylated primer: forward 5′- gtt ttt tcg agt tgg tat ttg ttc -3′, reverse 5′- aac ccc ata aat aaa ttt cta cgt a -3′; unmethylated primer: forward 5′- ttt ttt gag ttg gta ttt gtt tgg -3′; reverse 5′- aac ccc ata aat aaa ttt cta cat a -3′. Product size: 166 bp (methylated) and 164 bp (unmethylated); CpG island size: 231 bp; melting temperature (Tm): 63.8°C. The final results for DNA methylation were normalized using unmethylated results as input [45].

### *In vivo* rat experiments

The animal protocol conformed to US NIH guidelines (Guide for the Care and Use of Laboratory Animals, No. 85–23, revised 1996) and was reviewed and approved by the Institutional Animal Care and Use Committee.

#### Rat protocol 1: generation of diabetic rats

Two-month-old rats were fed a high-fat diet for 1 month followed by injection of 35 mg/kg streptozotocin (STZ) after an 8-h fast. Blood glucose levels were monitored for 4 weeks continuously and rats with blood glucose levels >11.1 mM were selected as the diabetes positive group. The control (CTL) group received only vehicle (VEH) injection [24,46].

#### Rat protocol 2: induction of cutaneous burn injury

The cutaneous burn injury was introduced to rats in protocol 1. The dorsa of the rats were exposed to a hot copper pillar (2-cm diameter) at 75°C for 15 s and the subsequent wound healing process was monitored and evaluated [24,46].

#### Rat protocol 3: BA treatment of cutaneous burns in a diabetic rat model

The rats in protocol 2 received either VEH or BA administration. In the procedure for IP administration, BA was dissolved in 0.1% Dimethylsulfoxide (DMSO) (diluted by 0.9% NaCl solution) and administered intraperitoneally every 3 days at a dose of 10 mg/kg body weight for 4 weeks starting from 1 week before the introduction of burn injury. For TOP administration, the dissolved BA was administrated by spraying in a dose of 20 μM each day continuously for 3 weeks starting from the second day after the introduction of burn injury. The experimental rats were randomly separated into four groups as follows: group 1: CTL rats with VEH treatment (CTL/VEH); group 2: STZ-induced diabetic (STZ) rats with VEH treatment (STZ/VEH); group 3: STZ rats with BA TOP administration (STZ/BA-TOP); group 4: STZ rats with BA IP administration (STZ/BA-IP); group 5: STZ rats with BA treatment by both TOP and IP administration (STZ/BA-IP/TOP). Wound status was monitored and evaluated throughout the treatment. After treatment, the animals were subjected to glucose/insulin tolerance tests. Whole blood was then withdrawn by heart puncture and the serum was prepared by centrifugation. Peripheral blood mononuclear cells (PBMCs) were isolated from the blood using Ficoll-Paque Plus lymphocyte separation medium and fibroblast cells were isolated from the underarm area of skin from treated mice for *in vitro* biological assays [47]. The rats were sacrificed and the wound tissues and soleus muscles were collected.

### Analysis of glucose uptake

Treated cells and the soleus muscles were used for this assay. The soleus muscles were isolated from treated rats and were dissected, weighed, pre-incubated (30 min) and then incubated (60 min) at 37°C in Krebs Ringer–bicarbonate buffer with a composition of 122 mM NaCl, 3 mM KCl, 1.2 mM MgSO_4_, 1.3 mM CaCl_2_, 0.4 mM KH_2_PO_4_ and 25 mM NaHCO_3_ and bubbled with O_2_/CO_2_ (95:5%, v/v) until pH 7.4. [U-^14^C]-2-Deoxy-D-glucose (^14^C-DG) (0.1 μCi/mL) was added to each sample during the incubation. After incubation, the muscles were placed in screw-cap tubes containing 1 mL of distilled water and frozen at −20°C in a freezer followed by 10 min of boiling. Aliquots of tissue and external medium (25 μL) were placed in a scintillation counter for radioactivity measurements [48] and the glucose uptake results were expressed as counts per min/ml of incubation medium [31].

### Intraperitoneal glucose tolerance test

For the glucose tolerance test, pre-treated mice were injected intraperitoneally with 2 g/kg body weight of glucose after a 6-hour fasting period. Blood samples were collected from the tail vein and blood glucose was monitored using a OneTouch Ultra®2 Glucometer at time points of 0, 15, 30, 60, 90 and 120 min after injection. Serum insulin levels were evaluated using the rat insulin enzyme-linked immunosorbent assay (ELISA) kit (#ERINS from Invitrogen) according to manufacturer’s instructions [34,49].

### Measurement of oxidative stress

The Glutathione/Glutathione disulfide (GSH/GSSG) ratio was determined using the GSH/GSSG ratio detection assay kit (fluorometric - green) (#ab205811 from Abcam) and the transcriptional activity of Nrf2 was determined using the Nrf2 transcription factor assay kit (colorimetric) (#ab207223 from Abcam). Methylglyoxal (MG) formation was determined using the methylglyoxal assay kit (#ab241006 from Abcam) and 3-nitrotyrosine formation was measured using the 3-nitrotyrosine ELISA kit (#ab116691 from Abcam) according to the manufacturer’s instructions.

### Statistical analysis

One-way Analysis of variance (ANOVA) analysis together with the Bonferroni *post hoc* test was employed to evaluate statistical significance among the treatments under normal distribution. The experiments were repeated at least four times (*n* = 4) unless otherwise mentioned. The data are presented as mean ± standard deviation (SD) and SPSS 22 software was employed for the analysis of statistical difference. *P* value <0.05 indicates a significant difference [24,46].

**Figure 1.**
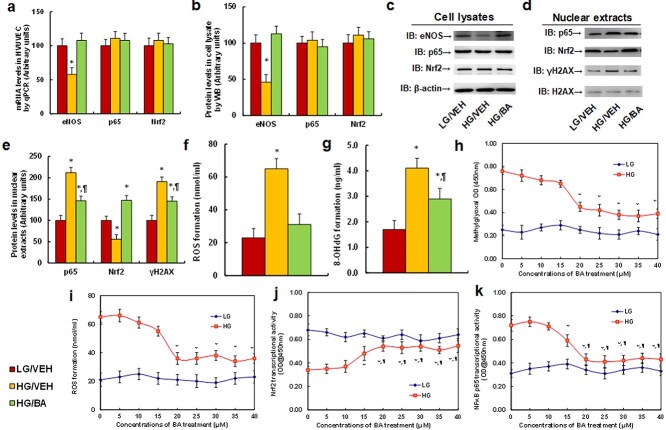
BA ameliorates hyperglycemia-induced oxidative stress and inflammation in HUVECs. (**a**–**g**) HUVECs were treated with low glucose (LG, 5 mM), high glucose (HG, 25 mM) or HG plus 20 μM BA for 4 days, and the cells were harvested for biological assays. (a) mRNA levels by qPCR, *n* = 4. (b) Protein quantitation in cell lysates by western blot, *n* = 5. (c) Representative pictures of western blots for (b). (d) Representative pictures of western blots for nuclear extracts. (e) Protein quantitation by western blot for (d), *n* = 5. (f) ROS formation, *n* = 5. (g) 8-OHdG formation, *n* = 5. ^*^p < 0.05 *vs* LG/VEH group; ^¶^*p* < 0.05 *vs* HG/VEH group. (**h**–**k**) HUVECs were treated by LG, HG or HG plus different concentrations of BA (from 0–40 μM) for biological assays at 24 h. (h) Methylglyoxal formation. (i) ROS formation. (j) Nrf2 transcriptional activity. (k) NFκB p65 transcriptional activity. *n* = 4. ^*^p < 0.05 *vs* 0 μM BA group; ^¶^*p* < 0.05 *vs* 15 μM BA group. Data are expressed as mean ± SD. *BA* betulinic acid, *HUVECs* human umbilical vein endothelial cells, *ROS* reactive oxygen species, *Nrf2* nuclear factor erythroid 2-related factor 2, *8-OHdG* 8-Hydroxy-2-deoxyguanosine, *NFκB* nuclear factor κB, *VEH* vehicle, *WB* western blot, *SD* standard deviation, *OD* optical density

## Results

### BA ameliorates hyperglycemia-induced oxidative stress and inflammation in human umbilical vein endothelial cells (HUVECs)

We determined the potential effect of BA and hyperglycemia on oxidative stress and inflammation. The HUVECs were incubated with 5 mM glucose (low glucose, LG), 25 mM glucose (high glucose, HG) or HG plus 20 μM BA for 4 days, and the cells were harvested for biological assays. Our results showed that HG treatment significantly decreased mRNA (Figure 1a) and protein levels (Figures 1b, c and S1a, see online supplementary material) of eNOS compared to the LG group, but had no effect on Nrf2 or NFκB p65. Additionally, BA treatment (HG/BA) completely reversed the HG-mediated effect. Moreover, HG treatment significantly increased NFκB p65 activity and phospho-Ser139 histone H2AX (γH2AX) formation, but decreased Nrf2 activity compared to the LG group. BA treatment (HG/BA) either partly or completely reversed this effect (Figures 1d, e and S1b, see online supplementary material). We also measured oxidative stress, and the results showed that BA treatment partly reversed hyperglycemia-mediated increased reactive oxygen species (ROS) formation (Figure 1f) and 8-hydroxy-2′-deoxyguanosine (8-OHdG) formation (Figure 1g). We then established dose–response curves for the effect of BA on hyperglycemia-mediated oxidative stress and inflammation, and the results showed that BA (20 μM) treatment significantly ameliorated hyperglycemia (HG)-mediated increased MG formation (Figure 1h) and ROS formation (Figure 1i), as well as hyperglycemia (HG)-mediated decreased Nrf2 activity (Figure 1j) and increased NFκB p65 activity (Figure 1k). We conclude that BA treatment ameliorates hyperglycemia-mediated oxidative stress and inflammation in HUVECs.

**Figure 2.**
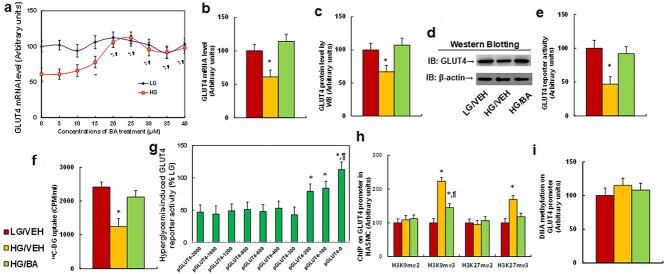
BA reverses hyperglycemia-mediated GLUT4 suppression by modulation of histone methylation on the GLUT4 promoter in HASMCs. (**a**–**f**) HASMCs were treated with low glucose (LG, 5 mM), high glucose (HG, 25 mM) or HG plus 20 μM BA for 4 days, and the cells were harvested for biological assays. (a) Dose–response curve of BA on GLUT4 mRNA levels, *n* = 4. ^*^p < 0.05 *vs* 0 μM BA group; ^¶^*p* < 0.05 *vs* 15 μM BA group. (b) mRNA levels by qPCR, *n* = 4. (c) Protein quantitation by western blot, *n* = 5. (d) Representative pictures of western blots for (c). (e) GLUT4 reporter assay, *n* = 5. (f) ^14^C-DG uptake assay, *n* = 5. ^*^p < 0.05 *vs* LG/VEH group. (**g**) The conditionally immortalized HASMCs were transfected by either GLUT4 full length (pGLUT4–2000) or deletion plasmids for 24 h; the cells were then treated with either LG or HG for 3 days and HG-mediated relative GLUT4 reporter activities (% LG) were calculated, *n* = 4. ^*^p < 0.05 *vs* pGLUT4–2000 group. (**h**,**i**) Treated cells from (a) were used for biological assays. (h) ChIP analysis on GLUT4 promoter. *n* = 4, *p* < 0.05 *vs* LG/VEH group; ^¶^*p* < 0.05 *vs* HG/VEH group. (i) DNA methylation assay on the GLUT4 promoter. Data are expressed as mean ± SD. *BA* betulinic acid, *HAMSCs* human primary aorta smooth muscle cells, *GLUT4* glucose transporter type 4, *VEH* vehicle, *SD* standard deviation

### BA reverses hyperglycemia-induced GLUT4 suppression by modulation of histone methylation on the GLUT4 promoter

We determined the molecular effect of BA and hyperglycemia on GLUT4 expression in HASMCs and found that 15 μM BA partly, while 20 μM of BA completely, reversed hyperglycemia-mediated decreased expression of GLUT4 mRNA (Figure 2a, b) and protein (Figures 2c, d and S1c, see online supplementary material) as well as hyperglycemia-mediated decreased GLUT4 reporter activity (Figure 2e) and ^14^C-DG uptake (Figure 2f). We then investigated the possible mechanism for hyperglycemia-mediated GLUT4 suppression. A series of progressive 5′-promoter deletions for GLUT4 reporter constructs was generated, and either GLUT4 full length (pGLUT4–2000) or deletion reporter plasmids were transiently transfected into conditionally immortalized HASMCs. Reporter activity was then measured after a 3-day treatment with either LG or HG. We found that hyperglycemia-mediated GLUT4 suppression occurred in the GLUT4 deletion constructs of pGLUT4–2000, −1600, −1400, −1200, −800, −600 and −400, while reporter suppression was partly restored in pGLU4–200 and −100 and completely restored in the pGLUT4–0 construct, suggesting that hyperglycemia-mediated transcriptional suppression is located in the range of −300 ~ −0 on the GLUT4 promoter (Figure 2g). We then evaluated possible epigenetic changes in this area, and results showed that HG treatment significantly increased histone methylation at H3K9me3 and H3K27me3 compared to LG treatment and that BA treatment partly reversed this effect (Figure 2h). On the other hand, there was no difference among treatments in histone methylation at H3K9me2 or H3K27me2 or in DNA methylation on the GLUT4 promoter (Figure 2i). Additionally, we evaluated possible epigenetic changes on histone 4 methylation (Figure S2a, see online supplementary material) and histone 3 acetylation (Figure S2b, see online supplementary material), and the results showed no effect among the treatments. We conclude that BA treatment partly reverses hyperglycemia-mediated GLUT4 suppression by modulation of histone methylation on the GLUT4 promoter.

### BA administration ameliorates diabetes-induced oxidative stress and inflammation in PBMCs

**Figure 3.**
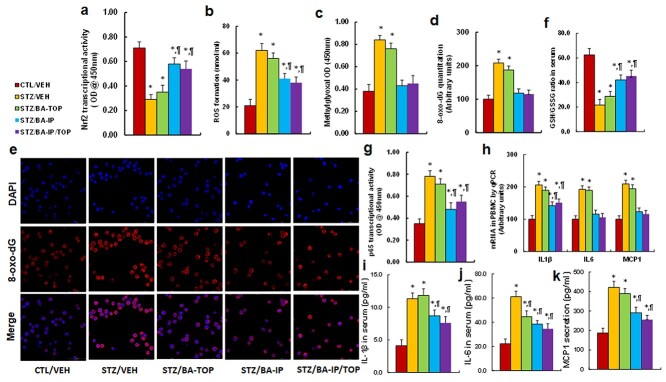
BA administration ameliorates hyperglycemia-induced oxidative stress and inflammation in PBMCs. Rat models with cutaneous burn injury received either CTL or STZ treatment and then received treatment of either vehicle (VEH) or BA administration through intraperitoneal (IP), topical (TOP) or both methods (IP/TOP); the serum and/or PBMCs were then isolated on day 23 after introduction of burn injury for biological assays. (**a**) Nrf2 transcriptional activity, *n* = 5. (**b**) ROS formation, *n* = 5. (**c**) Methylglyoxal formation, *n* = 5. (**d**) Quantitation of 8-oxo-dG formation, *n* = 5. (**e**) Representative pictures of 8-oxo-dG staining (red) and DAPI staining (blue) for (d). (**f**) GSH/GSSG ratio in serum, *n* = 5. (**g**) NFκB p65 transcriptional activity, *n* = 5. (**h**) mRNA level in PBMC, *n* = 4. (**i**) serum IL-1β level, *n* = 5. (**j**) serum IL-6 level, *n* = 5. (**k**) serum MCP1 level, *n* = 5. ^*^p < 0.05 *vs* CTL/VEH group; ^¶^*p* < 0.05 *vs* STZ/VEH group. Data are expressed as mean ± SD. *BA* betulinic acid, *PBMCs* peripheral blood mononuclear cells, *CTL* control, *STZ* streptozotocin, *DAPI* 4,6-diamidino-2-phenylindole, *IL* interleukin, *MCP1* monocyte chemoattractant protein-1, *SD* standard deviation, *OD* optical density

We determined the potential effect of BA and diabetes on oxidative stress in PBMCs. Diabetic rats with burn injury received BA administration by IP, TOP or both treatments (IP/TOP), and then the serum and/or PBMCs were isolated for biological assays. We found that the diabetes (STZ/VEH) group showed significantly reduced Nrf2 transcriptional activity in PBMCs compared to the CTL/VEH group, and that TOP administration of BA (BA-TOP) had little effect, while BA administration by either IP alone or a combination of IP and TOP (IP/TOP) partly reversed this effect (Figure 3a). Additionally, STZ/VEH treatment significantly potentiated ROS generation (Figure 3b), MG formation (Figure 3c) and 8-oxo-dG formation (Figure 3d, e) in PBMCs compared to the CTL/VEH group. BA-TOP had little effect, while treatment with either BA-IP or BA-IP/TOP significantly reversed this effect. We also evaluated the GSH/GSSG ratio in serum, and the results showed that treatment with either BA-IP or BA-IP/TOP partly reversed diabetes (STZ/VEH)-mediated reduction of the GSH/GSSG ratio, while the BA-TOP group showed no effect (Figure 3f). We then evaluated the effect of BA on diabetes-mediated inflammation and found that STZ/VEH treatment significantly potentiated NFκB p65 transcriptional activity in PBMCs compared to the CTL/VEH group; BA-TOP treatment showed little effect, while treatment with either BA-IP or BA-IP/TOP partly reversed this effect (Figure 3g). We also determined the mRNA levels of pro-inflammatory cytokines and found that treatment with BA-IP or BA-IP/TOP either partly or completely reversed diabetes (STZ/VEH)-induced increased cytokine expression, while BA-TOP treatment showed little effect (Figure 3h). We also determined the serum levels of the pro-inflammatory cytokines, including interleukin-1β (IL-1β) (Figure 3i), IL-6 (Figure 3j) and monocyte chemoattractant protein-1 (MCP1) (Figure 3k), and the expression levels were similar to that of related mRNA levels. We conclude that intraperitoneal administration of BA ameliorates diabetes-induced oxidative stress and inflammation in PBMCs.

**Figure 4.**
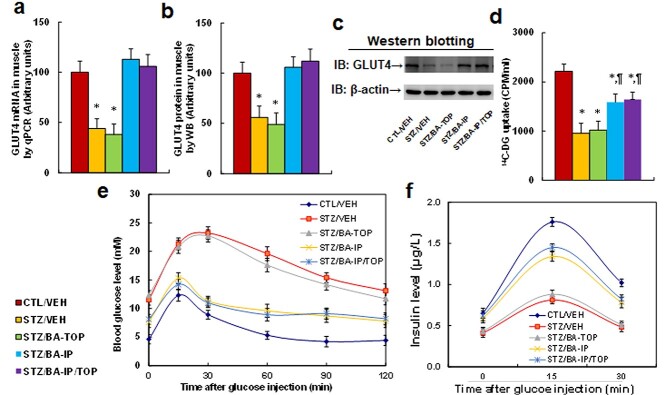
BA administration ameliorates diabetes-mediated GLUT4 suppression and glucose intolerance in muscles. Rat models with cutaneous burn injury received CTL or STZ treatment and then received treatment of either vehicle (VEH) or BA administration by intraperitoneal (IP), topical (TOP) or both methods (IP/TOP). The tissues or soleus muscles were isolated and used for analysis on day 23 after burn injury. (**a**–**d**) The muscles were used for biological assays. (a) mRNA levels, *n* = 4. (b) Quantitation of protein level by western blot, *n* = 5. (c) Representative western blots for (b). (d) ^14^C-DG uptake assay, *n* = 5. ^*^p < 0.05 *vs* LG/VEH group; ^¶^*p* < 0.05 *vs* STZ/VEH group. (**d**,**e**) Rats were used for the intraperitoneal glucose tolerance test. (e) Blood glucose levels, *n* = 5. (f) Serum insulin levels, *n* = 5. Data are expressed as mean ± SD. *BA* betulinic acid, *GLUT4* glucose transporter type 4, *CTL* control, *STZ* streptozotocin, *LG* low glucose, *SD* standard deviation

### BA administration ameliorates diabetes-mediated GLUT4 suppression and glucose intolerance in muscles

We determined the effect of BA on diabetes-mediated GLUT4 suppression in muscles and found that STZ/VEH treatment significantly reduced GLUT4 expression of both mRNA (Figure 4a) and protein levels (Figures 4b, c and S1d, see online supplementary material) compared to the CTL/VEH group. BA-TOP treatment showed little effect, while treatment with either BA-IP or BA-IP/TOP completel reversed this effect. Additionally, treatment with either BA-IP or BA-IP/TOP partly reversed diabetes (STZ/VEH)-mediated suppression of ^14^C-DG uptake, and again BA-TOP treatment had little effect (Figure 4d). We also evaluated the potential effect of BA on fibroblast cells isolated from the skin of treated mice and found that BA treatment completely reversed diabetes (STZ)-mediated GLUT4 suppression (Figure S3a, see online supplementary material), partly reversed diabetes-induced epigenetic changes on the GLUT4 promoter (Figure S3b), and completely reversed diabetes-mediated glucose uptake (Figure S3c). Finally, we determined the possible effect of BA administration on diabetes-mediated glucose intolerance and found that STZ/VEH treatment significantly increased blood glucose levels after glucose injection during the intraperitoneal glucose tolerance test compared to the CTL/VEH group. BA-TOP treatment showed little effect, while treatment with either BA-IP or BA-IP/TOP partly reversed this effect (Figure 4e). We again determined the insulin levels and found that the diabetic (STZ/VEH) group had significantly less insulin secretion during the intraperitoneal glucose tolerance test compared to the CTL/VEH group. BA-TOP treatment showed little effect, while treatment with either BA-IP or BA-IP/TOP partly reversed this effect (Figure 4f). We conclude that BA-IP administration has significant anti-diabetic effects on diabetes-mediated GLUT4 suppression and glucose intolerance.

### BA administration ameliorates diabetes-mediated oxidative stress and inflammation

**Figure 5.**
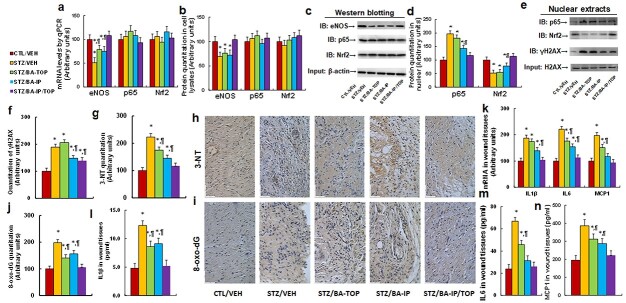
BA administration ameliorates diabetes-mediated oxidative stress and inflammation in wound tissues. Rat models with cutaneous burn injury received CTL or STZ treatment and then received treatment of either vehicle (VEH) or BA administration by intraperitoneal (IP), topical (TOP) or both methods (IP/TOP); the wound tissues were then isolated on day 23 after burns for biological assays. (**a**) mRNA levels by qPCR, *n* = 4. (**b**) Protein quantitation by western blot, *n* = 5. (**c**) Representative western blots for (b). (**d**) Protein quantitation in nuclear extracts by western blot, *n* = 5. (**e**) Representative western blots for (d). (**f**) Quantitation of γH2AX levels for (e). (**g**) 3-Nitrotyrosine (3-NT) quantitation, *n* = 5. (**h**) Representative pictures of 3-NT staining for (g). (**i**) 8-oxo-dG quantitation, *n* = 5. (**j**) Representative pictures of 8-oxo-dG staining for (i). (**k**) mRNA level in wound tissues, *n* = 4. (**l**) IL-1β in tissues, *n* = 5. (**m**) IL-6 in tissues, *n* = 5. (**n**) MCP1 in tissues, *n* = 5. ^*^p < 0.05 *vs* CTL/VEH group; ^¶^*p* < 0.05 *vs* STZ/VEH group. Data are expressed as mean ± SD. *BA* betulinic acid, *γH2AX* PhosphoSer139 Histone H2AX, *CTL* control, *STZ* streptozotocin, *MCP1* monocyte chemoattractant protein-1, *IL* interleukin, *SD* standard deviation

We determined the effect of BA and hyperglycemia on gene expression in wound tissues. We found that STZ/VEH treatment significantly decreased mRNA (Figure 5a) and protein levels (Figures 5b, c and S1e, see online supplementary material) of eNOS in whole cell lysates compared to the CTL/VEH group; treatment with either BA-TOP or BA-IP partly, while BA-IP/TOP treatment completely, reversed this effect. Additionally, there was no difference in the expression of Nrf2 and NFκB p65 among all the treatments. We then determined the transcriptional activity of Nrf2 and NFκB p65 by measuring related protein levels in nuclear extracts, and the results from western blotting showed that treatments with BA-TOP and BA-IP partly, while BA-IP/TOP treatment completely, reversed diabetes (STZ/VEH)-mediated NFkB p65 activation and Nrf2 suppression (Figures 5d, e and S1f, see online supplementary material). We evaluated the effect of BA and diabetes on oxidative stress, and the results showed that treatment with BA-TOP and BA-IP partly, while BA-IP/TOP treatment almost completely, reversed diabetes (STZ/VEH)-mediated increased γH2AX formation (Figures 5e, f and S1f), 3-nitrotyrosine formation (Figure 5g, h) and 8-oxo-dG formation (Figure 5i, j). We also determined the effect of BA and diabetes on inflammation in wound tissues, and the results showed that diabetes (STZ/VEH) treatment significantly increased mRNA levels of IL1β, IL6 and MCP1; treatment with BA-TOP and BA-IP partly, while BA-IP/TOP treatment completely, reversed this effect (Figure 5k). We also evaluated protein levels from wound tissues for the pro-inflammatory cytokines, including IL1β (Figure 5l), IL6 (Figure 5m) and MCP1 (Figure 5n), and the expression was similar to related mRNA levels. We conclude that BA administration ameliorates hyperglycemia-mediated oxidative stress and inflammation.

**Figure 6.**
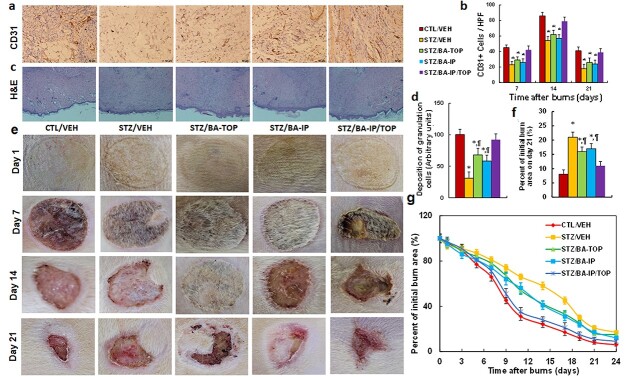
BA administration accelerates diabetic wound healing. Rat models with cutaneous burn injury received CTL or STZ treatment and then received treatment of either vehicle (VEH) or BA administration by intraperitoneal (IP), topical (TOP) or both methods (IP/TOP). The wounds were then isolated for analysis and the healing process was evaluated. (**a**) Representative pictures for evaluation of vascularity (assessed by CD31 immunohistochemistry). (**b**) Quantitative numbers of CD31 positive per HPF area on day 14 after burns for (a), *n* = 8. (**c**) H&E staining of granulation cells on day 21 after burns. (**d**) Granulation cell deposition from (c), *n* = 8. (**e**) Photographs of representative wounds on days 1, 7, 14 and 21 after burns. (**f**) Quantitation of burn area on day 21, *n* = 8. (**g**) Graphical depiction of wound areas on different days after burns, *n* = 8. *p* < 0.05 *vs* CTL/VEH group; ^¶^*p* < 0.05 *vs* STZ/VEH group. Data are expressed as mean ± SD. *BA* betulinic acid, *CTL* control, *STZ* streptozotocin, *H&E* hematoxylin and eosin, *HPF* high power field, *SD* standard deviation

**Figure 7.**
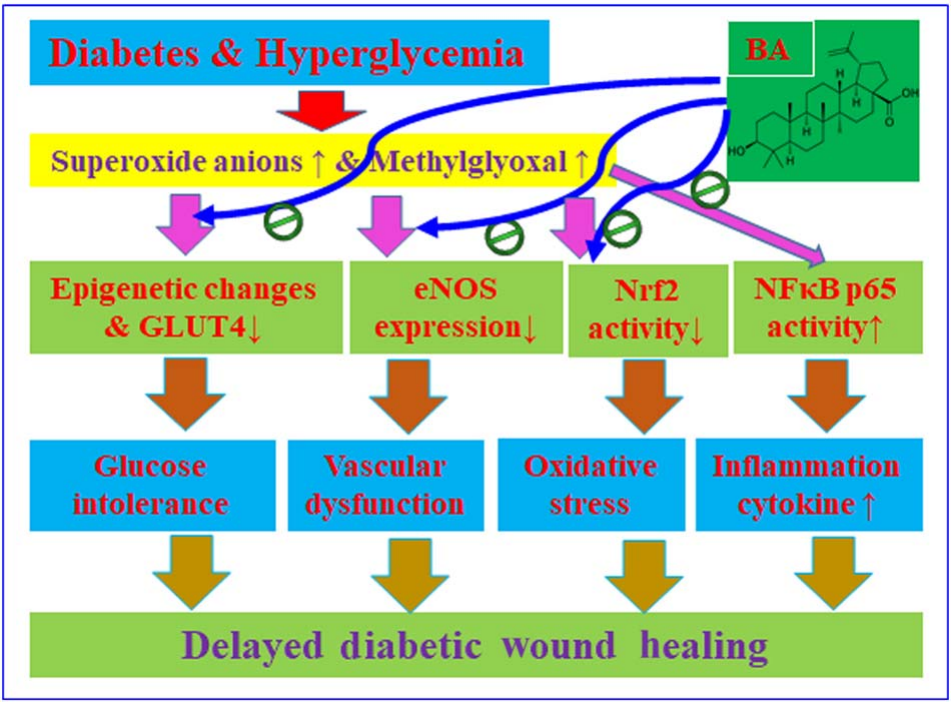
Schematic model for BA-mediated acceleration of diabetic wound healing. *BA* Betulinic acid; *eNOS* endothelial nitric oxide synthase, *GLUT4* glucose transporter type 4, *NFκB p65* nuclear factor NFκB p65 subunit, *Nrf2* nuclear factor erythroid 2-related factor 2

### BA administration accelerates diabetic wound healing

We determined the effect of BA administration on diabetic wound healing. The CD31 positive cells in wound tissues were identified by immunohistochemistry on days 7, 14 and 21, respectively after burns, and we found that diabetes (STZ/VEH) significantly reduced the generation of CD31 positive cells compared to the CTL/VEH group; treatment with either BA-TOP or BA-IP showed little effect, while this effect was completely reversed in the BA-IP/TOP group (Figure 6a, b). We then determined the deposition of granulation cells by hematoxylin and eosin (H&E) staining and found that treatment with either BA-TOP or BA-IP partly, while BA-IP/TOP treatment completely, reversed diabetes-mediated decreased deposition of granulation cells (Figure 6c, d). Finally, we evaluated the effect of BA administration on the progress of diabetic wound healing. Pictures of the wound were taken every day after burns, and the results indicated that treatment with either BA-TOP or BA-IP partly, and BA-IP/TOP treatment completely, reversed diabetes-mediated delayed diabetic wound healing (Figure 6e–g).

### Schematic model for BA-mediated acceleration of diabetic wound healing

Diabetes or hyperglycemia-mediated overgeneration of superoxide anions and MG formation results in decreased expression of eNOS, epigenetic changes and GLUT4 suppression, as well as suppressed Nrf2 transcriptional activity and increased NFκB p65 activity, subsequently triggering oxidative stress, inflammation, vascular dysfunction and glucose intolerance. On the other hand, BA administration can either partly or completely reverse those diabetes-mediated effects; thus, BA administration could significantly accelerate diabetic wound healing (Figure 7).

## Discussion

In this study, we demonstrate that BA reverses hyperglycemia-mediated GLUT4 suppression in HASMCs and also partly reverses hyperglycemia-mediated eNOS suppression, Nrf2 suppression and NFκB p65 activation in HUVECs. Additionally, BA administration in rats ameliorates diabetes-mediated glucose intolerance in muscles and fibroblasts, as well as diabetes-induced oxidative stress and inflammation in the circulation and wound tissues. Finally, we show that administration of BA by both IP and TOP methods significantly accelerates diabetic wound healing.

### BA-mediated effect on oxidative stress and inflammation

It has been reported that BA attenuates oxidative stress by activation of the Nrf2 signaling pathway [23,50]. Our results showed that BA treatment significantly increased Nrf2 transcriptional activity but showed no effect on Nrf2 expression [25], subsequently ameliorating hyperglycemia-mediated ROS generation, 8-oxo-dG formation and MG formation, and decreased the GSH/GSSG ratio in the circulatory system. Additionally, our results showed that BA treatment significantly ameliorated hyperglycemia-mediated NFκB p65 transcriptional activation [51], subsequently decreasing the release of pro-inflammatory cytokines, including IL1β, IL6 and MCP1 [24]. Taken together, BA can ameliorate hyperglycemia-induced oxidative stress and inflammation; this effect may partly contribute to its beneficial effect on diabetic wound healing, athough the detailed mechanism remains largely unknown.

### BA-mediated anti-diabetic effect

It has been reported that BA induces GLUT4 activation in muscle cells [31]. A BA-mediated anti-diabetic effect has been previously reported [32–35], although the related mechanism remains largely unknown. Our results showed that BA treatment increased GLUT4 expression in both muscle tissue and fibroblasts, subsequently ameliorating diabetes-mediated glucose intolerance. In this scenario, BA could be a potential candidate for treatment of diabetes and could subsequently be beneficial for therapeutic treatment of diabetic wound healing by ameliorating related diabetic complications [52,53].

### BA-mediated beneficial effect on diabetic wound healing

In addition to the beneficial effect of BA on diabetes-mediated oxidative stress, inflammation and glucose intolerance, our results also showed that BA treatment significantly reversed hyperglycemia-mediated eNOS suppression [22], indicating that BA may play a vascular protective role in diabetic complications [54]. In this study, BA was administrated through either IP or TOP methods for the treatment of burn injury in diabetic rats, and the results showed that BA administration by IP significantly attenuated diabetes-mediated oxidative stress and inflammation in PBMCs, while BA administration by TOP showed little effect. On the other hand, IP administration of BA had a significantly lower effect on wound tissues compared to TOP administration; this can be explained as BA has very low gastrointestinal absorption. Finally, our results showed that BA administration by both IP and TOP significantly accelerated diabetic wound healing; this suggests that treatment of diabetic wound healing should not just focus on the wound/injury itself; instead, more attention should be paid to diabetes and diabetes-mediated complications and dysfunction. This is the first time that BA has been used for the treatment of diabetic burn injury with positive results due to its anti-diabetic effect and potential protective role in diabetes-mediated dysfunction, such as oxidative stress and inflammation [55,56].

## Conclusions

BA treatment ameliorates diabetes-mediated glucose intolerance by activation of GLUT4 expression, attenuates hyperglycemia-induced oxidative stress and inflammation by Nrf2 activation and NFκB p65 signaling suppression, and plays a vascular protective role by stimulation of eNOS expression. BA administration by both IP and TOP methods significantly accelerates diabetic wound healing. We conclude that BA accelerates diabetic wound healing by modulating hyperglycemia-induced oxidative stress, inflammation and glucose intolerance and that BA may be a promising therapeutic candidate for the treatment of diabetic wound healing.

**Abbreviations**
BA: Betulinic acid; CTL: Control; ^14^C-DG: [U-^14^C]-2-Deoxy-D-glucose; eNOS: Endothelial nitric oxide synthase; GLUT4: Glucose transporter type 4; HAMSC: Human primary aorta smooth muscle cells; HG: High glucose; γH2AX: Phospho-Ser139 Histone H2AX; IL1β: Interleukin-1β; IP: Intraperitoneal; LG: Low glucose; MCP1: Monocyte chemoattractant protein-1; MG: Methylglyoxal; NFκB p65: Nuclear factor NFκB p65 subunit; Nrf2: Nuclear factor erythroid 2-related factor 2; 8-OHdG: 8-Hydroxy-2′-deoxyguanosine; PBMC: Peripheral blood mononuclear cells; ROS: Reactive oxygen species; STZ: Streptozotocin; VEH: Vehicle; TOP: topical.

## Funding

This study was financially supported by The National Natural Science Foundation of China, Project #: 81772097 and Revival Program of Growth Factors, Project #: SZYZ-TR-10.

## Authors’ contributions

PY wrote the paper, PY and WX designed, analyzed the data and interpreted the experiments. WH, ZH and XH performed part of the animal experiments, HZ performed part of the gene expression analysis. ML performed statistical analysis. WX performed the remaining experiments. All authors read and approved the final manuscript.

## Ethics approval and consent to participate

The animal protocol conformed to US NIH guidelines (Guide for the Care and Use of Laboratory Animals, No. 85–23, revised 1996), and was reviewed and approved by the Institutional Animal Care and Use Committee.

## Conflicts of interest

None declared.

## Supplementary Material

BA-220119-SI_tkac007

## References

[1] Rodrigues M , Kosaric N, Bonham CA, Gurtner GC. Wound healing: a cellular perspective. Physiol Rev. 2019;99:665–706. 30475656 10.1152/physrev.00067.2017 PMC6442927

[2] King A , Balaji S, Keswani SG, Crombleholme TM. The role of stem cells in wound angiogenesis. Adv Wound Care (New Rochelle). 2014;3:614–25. 25300298 10.1089/wound.2013.0497 PMC4183912

[3] Cerqueira MT , Pirraco RP, Marques AP. Stem cells in skin wound healing: are we there yet? Adv Wound Care (New Rochelle). 2016;5:164–75. 27076994 10.1089/wound.2014.0607 PMC4817598

[4] Xie W , Zhou X, Hu W, Chu Z, Ruan Q, Zhang H, et al. Pterostilbene accelerates wound healing by modulating diabetes-induced estrogen receptor beta suppression in hematopoietic stem cells. Burns Trauma. 2021;9:tkaa045. 10.1093/burnst/tkaa045. 33654697 PMC7901710

[5] McInnes RL , Cullen BM, Hill KE, Price PE, Harding KG, Thomas DW, et al. Contrasting host immuno-inflammatory responses to bacterial challenge within venous and diabetic ulcers. Wound Repair Regen. 2014;22:58–69. 24354589 10.1111/wrr.12133

[6] El-Osta A , Brasacchio D, Yao D, Pocai A, Jones PL, Roeder RG, et al. Transient high glucose causes persistent epigenetic changes and altered gene expression during subsequent normoglycemia. J Exp Med. 2008;205:2409–17. 18809715 10.1084/jem.20081188 PMC2556800

[7] Zhang QR , Yang X, Li Z, Jia JZ, Luo GX, Yu YL, et al. Effects of reactive oxygen species-responsive antibacterial microneedles on the full-thickness skin defect wounds with bacterial colonization in diabetic mice. Zhonghua Shao Shang Za Zhi. 2021;37:1024–35. 34794254 10.3760/cma.j.cn501120-20210831-00299 PMC11917343

[8] Esposito K , Nappo F, Marfella R, Giugliano G, Giugliano F, Ciotola M, et al. Inflammatory cytokine concentrations are acutely increased by hyperglycemia in humans: role of oxidative stress. Circulation. 2002;106:2067–72. 12379575 10.1161/01.cir.0000034509.14906.ae

[9] Thangarajah H , Yao D, Chang EI, Shi Y, Jazayeri L, Vial IN, et al. The molecular basis for impaired hypoxia-induced VEGF expression in diabetic tissues. Proc Natl Acad Sci U S A. 2009;106:13505–10. 19666581 10.1073/pnas.0906670106 PMC2726398

[10] Ceradini DJ , Yao D, Grogan RH, Callaghan MJ, Edelstein D, Brownlee M, et al. Decreasing intracellular superoxide corrects defective ischemia-induced new vessel formation in diabetic mice. J Biol Chem. 2008;283:10930–8. 18227068 10.1074/jbc.M707451200 PMC2447622

[11] Falanga V . Wound healing and its impairment in the diabetic foot. Lancet. 2005;366:1736–43. 16291068 10.1016/S0140-6736(05)67700-8

[12] Long M , Rojo de la Vega M, Wen Q, Bharara M, Jiang T, Zhang R, et al. An essential role of NRF2 in diabetic wound healing. Diabetes. 2016;65:780–93. 26718502 10.2337/db15-0564 PMC4764153

[13] Singh N , Armstrong DG, Lipsky BA. Preventing foot ulcers in patients with diabetes. JAMA. 2005;293:217–28. 15644549 10.1001/jama.293.2.217

[14] Martin P , Nunan R. Cellular and molecular mechanisms of repair in acute and chronic wound healing. Br J Dermatol. 2015;173:370–8. 26175283 10.1111/bjd.13954 PMC4671308

[15] Zhang J , Yang P, Liu D, Gao M, Wang J, Yu T, et al. Inhibiting hyper-O-GlcNAcylation of c-Myc accelerate diabetic wound healing by alleviating keratinocyte dysfunction. *Burns*. Burns Trauma. 2021;9:tkab031. 10.1093/burnst/tkab031. PMC8499626 34646892

[16] Cao WB , Gao CY. Research advances on multifunctional hydrogel dressings for treatment of diabetic chronic wounds. Zhonghua Shao Shang Za Zhi. 2021;37:1090–8. 34794262 10.3760/cma.j.cn501120-20210715-00249 PMC11917229

[17] Yao D , Li H, Gou Y, Zhang H, Vlessidis AG, Zhou H, et al. Betulinic acid-mediated inhibitory effect on hepatitis B virus by suppression of manganese superoxide dismutase expression. FEBS J. 2009;276:2599–614. 19348625 10.1111/j.1742-4658.2009.06988.x

[18] Pisha E , Chai H, Lee IS, Chagwedera TE, Farnsworth NR, Cordell GA, et al. Discovery of betulinic acid as a selective inhibitor of human melanoma that functions by induction of apoptosis. Nat Med. 1995;1:1046–51. 7489361 10.1038/nm1095-1046

[19] Chintharlapalli S , Papineni S, Ramaiah SK, Safe S. Betulinic acid inhibits prostate cancer growth through inhibition of specificity protein transcription factors. Cancer Res. 2007;67:2816–23. 17363604 10.1158/0008-5472.CAN-06-3735

[20] Fulda S , Jeremias I, Debatin KM. Cooperation of betulinic acid and TRAIL to induce apoptosis in tumor cells. Oncogene. 2004;23:7611–20. 15361826 10.1038/sj.onc.1207970

[21] Yogeeswari P , Sriram D. Betulinic acid and its derivatives: a review on their biological properties. Curr Med Chem. 2005;12:657–66. 15790304 10.2174/0929867053202214

[22] Lee GH , Park JS, Jin SW, Pham TH, Thai TN, Kim JY, et al. Betulinic acid induces eNOS expression via the AMPK-dependent KLF2 Signaling pathway. J Agric Food Chem. 2020;68:14523–30. 33232606 10.1021/acs.jafc.0c06250

[23] Zhu L , Yi X, Ma C, Luo C, Kong L, Lin X, et al. Betulinic acid attenuates oxidative stress in the thymus induced by acute exposure to T-2 toxin via regulation of the MAPK/Nrf2 Signaling pathway. Toxins (Basel). 2020;12:540. 10.3390/toxins12090540. PMC7551141 32842569

[24] Li M , Yu H, Pan H, Zhou X, Ruan Q, Kong D, et al. Nrf2 suppression delays diabetic wound healing through sustained oxidative stress and inflammation. Front Pharmacol. 2019;10:1099. 31616304 10.3389/fphar.2019.01099 PMC6763603

[25] He F , Ru X, Wen T. NRF2, a transcription factor for stress response and beyond. Int J Mol Sci. 2020;21:4777. 10.3390/ijms21134777. PMC7369905 32640524

[26] Hosur V , Burzenski LM, Stearns TM, Farley ML, Sundberg JP, Wiles MV, et al. Early induction of NRF2 antioxidant pathway by RHBDF2 mediates rapid cutaneous wound healing. Exp Mol Pathol. 2017;102:337–46. 28268192 10.1016/j.yexmp.2017.03.003 PMC5436942

[27] Maher J , Yamamoto M. The rise of antioxidant signaling--the evolution and hormetic actions of Nrf2. Toxicol Appl Pharmacol. 2010;244:4–15. 20122947 10.1016/j.taap.2010.01.011

[28] Zhou Z , Choi JW, Shin JY, Kim DU, Kweon B, Oh H, et al. Betulinic acid ameliorates the severity of acute pancreatitis via inhibition of the NF-kappaB Signaling pathway in mice. Int J Mol Sci. 2021;22:6871. 10.3390/ijms22136871. PMC8268208 34206763

[29] Luo C , Huang C, Zhu L, Kong L, Yuan Z, Wen L, et al. Betulinic acid ameliorates the T-2 toxin-triggered intestinal impairment in mice by inhibiting inflammation and mucosal barrier dysfunction through the NF-kappaB Signaling pathway. Toxins (Basel). 2020;12:794. 10.3390/toxins12120794. PMC7763746 33322178

[30] Bailly C . Acankoreagenin and acankoreosides, a family of Lupane triterpenoids with anti-inflammatory properties: an overview. Ann N Y Acad Sci. 2021;1502:14–27. 34145915 10.1111/nyas.14623

[31] Castro AJ , Frederico MJ, Cazarolli LH, Bretanha LC, Tavares Lde C, Buss Zda S, et al. Betulinic acid and 1,25(OH)(2) vitamin D(3) share intracellular signal transduction in glucose homeostasis in soleus muscle. Int J Biochem Cell Biol. 2014;48:18–27. 24316531 10.1016/j.biocel.2013.11.020

[32] Ko BS , Kang S, Moon BR, Ryuk JA, Park S. A 70% ethanol extract of mistletoe rich in Betulin, Betulinic acid, and Oleanolic acid potentiated beta-cell function and mass and enhanced hepatic insulin sensitivity. Evid Based Complement Alternat Med. 2016;2016:7836823. 10.1155/2016/7836823. 26884795 PMC4738752

[33] Silva FS , Oliveira PJ, Duarte MF. Oleanolic, Ursolic, and Betulinic acids as food supplements or pharmaceutical agents for type 2 diabetes: promise or illusion? J Agric Food Chem. 2016;64:2991–3008. 27012451 10.1021/acs.jafc.5b06021

[34] Song TJ , Park CH, In KR, Kim JB, Kim JH, Kim M, et al. Antidiabetic effects of betulinic acid mediated by the activation of the AMP-activated protein kinase pathway. PLoS One. 2021;16:e0249109. 33819291 10.1371/journal.pone.0249109 PMC8021171

[35] Kumar S , Kumar V, Prakash O. Enzymes inhibition and antidiabetic effect of isolated constituents from Dillenia indica. Biomed Res Int. 2013;2013:382063. 10.1155/2013/382063. 24307994 PMC3838843

[36] Bildziukevich U , Ozdemir Z, Wimmer Z. Recent achievements in medicinal and supramolecular chemistry of Betulinic acid and its derivatives (double dagger). Molecules. 2019;24:3546. 10.3390/molecules24193546. PMC6803882 31574991

[37] Rios JL , Manez S. New pharmacological opportunities for Betulinic acid. Planta Med. 2018;84:8–19. 29202513 10.1055/s-0043-123472

[38] Kviecinski MR , David IM, Fernandes FS, Correa MD, Clarinda MM, Freitas AF, et al. Healing effect of Dillenia indica fruit extracts standardized to betulinic acid on ultraviolet radiation-induced psoriasis-like wounds in rats. Pharm Biol. 2017;55:641–8. 27951742 10.1080/13880209.2016.1266672 PMC6130706

[39] Martin ET , Kaye KS, Knott C, Nguyen H, Santarossa M, Evans R, et al. Diabetes and risk of surgical site infection: a systematic review and meta-analysis. Infect Control Hosp Epidemiol. 2016;37:88–99. 26503187 10.1017/ice.2015.249 PMC4914132

[40] Ruzicska E , Toth M, Tulassay Z, Somogyi A. Adrenomedullin and diabetes mellitus. Diabetes Metab Res Rev. 2001;17:321–9. 11747138 10.1002/dmrr.223

[41] Chung PY . Novel targets of pentacyclic triterpenoids in Staphylococcus aureus: a systematic review. Phytomedicine. 2020;73:152933. 10.1016/j.phymed.2019.152933. 31103429

[42] Ibrahim HA , Elgindi MR, Ibrahim RR, El-Hosari DG. Antibacterial activities of triterpenoidal compounds isolated from Calothamnus quadrifidus leaves. BMC Complement Altern Med. 2019;19:102. 10.1186/s12906-019-2512-x. 31072346 PMC6509848

[43] Bildziukevich U , Rarova L, Janovska L, Saman D, Wimmer Z. Enhancing effect of cystamine in its amides with betulinic acid as antimicrobial and antitumor agent in vitro. Steroids. 2019;148:91–8. 31022408 10.1016/j.steroids.2019.04.004

[44] Zhang H , Li L, Li M, Huang X, Xie W, Xiang W, et al. Combination of betulinic acid and chidamide inhibits acute myeloid leukemia by suppression of the HIF1α pathway and generation of reactive oxygen species. Oncotarget. 2017;8:94743–58. 29212263 10.18632/oncotarget.21889 PMC5706909

[45] Zou Y , Lu Q, Zheng D, Chu Z, Liu Z, Chen H, et al. Prenatal levonorgestrel exposure induces autism-like behavior in offspring through ERβ suppression in the amygdala. Mol Autism. 2017;8:46. 10.1186/s13229-017-0159-3. 28824796 PMC5561609

[46] Zhou X , Li M, Xiao M, Ruan Q, Chu Z, Ye Z, et al. ERβ accelerates diabetic wound healing by ameliorating Hyperglycemia-induced persistent oxidative stress. Front Endocrinol (Lausanne). 2019;10:499. 10.3389/fendo.2019.00499. 31396159 PMC6667639

[47] Seluanov A , Vaidya A, Gorbunova V. Establishing primary adult fibroblast cultures from rodents. J Vis Exp. 2010. 10.3791/2033 PMC3185624 20972406

[48] Cazarolli LH , Folador P, Moresco HH, Brighente IM, Pizzolatti MG, Silva FR. Mechanism of action of the stimulatory effect of apigenin-6-C-(2̋-O-alpha-l-rhamnopyranosyl)-beta-L-fucopyranoside on 14C-glucose uptake. Chem Biol Interact. 2009;179:407–12. 19070612 10.1016/j.cbi.2008.11.012

[49] Tahara A , Matsuyama-Yokono A, Nakano R, Someya Y, Shibasaki M. Effects of antidiabetic drugs on glucose tolerance in streptozotocin-nicotinamide-induced mildly diabetic and streptozotocin-induced severely diabetic mice. Horm Metab Res. 2008;40:880–6. 18819058 10.1055/s-0028-1087167

[50] Kong L , Zhu L, Yi X, Huang Y, Zhao H, Chen Y, et al. Betulinic acid alleviates spleen oxidative damage induced by acute intraperitoneal exposure to T-2 toxin by activating Nrf2 and inhibiting MAPK Signaling pathways. Antioxidants (Basel). 2021;10:158. 10.3390/antiox10020158. PMC7912660 33499152

[51] Wang S , Yang Z, Xiong F, Chen C, Chao X, Huang J, et al. Betulinic acid ameliorates experimental diabetic-induced renal inflammation and fibrosis via inhibiting the activation of NF-kappaB signaling pathway. Mol Cell Endocrinol. 2016;434:135–43. 27364889 10.1016/j.mce.2016.06.019

[52] Ahangarpour A , Shabani R, Farbood Y. The effect of betulinic acid on leptin, adiponectin, hepatic enzyme levels and lipid profiles in streptozotocin-nicotinamide-induced diabetic mice. Res Pharm Sci. 2018;13:142–8. 29606968 10.4103/1735-5362.223796 PMC5842485

[53] Yoon JJ , Lee YJ, Han BH, Choi ES, Kho MC, Park JH, et al. Protective effect of betulinic acid on early atherosclerosis in diabetic apolipoprotein-E gene knockout mice. Eur J Pharmacol. 2017;796:224–32. 27894808 10.1016/j.ejphar.2016.11.044

[54] Triggle CR , Ding H. A review of endothelial dysfunction in diabetes: a focus on the contribution of a dysfunctional eNOS. J Am Soc Hypertens. 2010;4:102–15. 20470995 10.1016/j.jash.2010.02.004

[55] Velnar T , Bailey T, Smrkolj V. The wound healing process: an overview of the cellular and molecular mechanisms. J Int Med Res. 2009;37:1528–42. 19930861 10.1177/147323000903700531

[56] Han G , Ceilley R. Chronic wound healing: a review of current management and treatments. Adv Ther. 2017;34:599–610. 28108895 10.1007/s12325-017-0478-y PMC5350204

