# Supplementary material for: Betulinic acid accelerates diabetic wound healing by modulating hyperglycemia-induced oxidative stress, inflammation and glucose intolerance
Source: Burns Trauma. 2022 Apr 9;10:tkac007. doi: 10.1093/burnst/tkac007 (PMC8993492; doi:10.1093/burnst/tkac007)
Supplement: BA-220119-SI_tkac007 [file ba-220119-si_tkac007.docx]

**Betulinic Acid Accelerates Diabetic Wound Healing by Modulating Hyperglycemia-Induced Oxidative Stress, Inflammation and Glucose Intolerance**

**Supplemental Information**

**Data S1: Materials and Methods**

Materials and Reagents. Human Primary Aorta Smooth Muscle Cells (HASMC, #PCS-100-012) were purchased from ATCC and maintained in Vascular Cell Basal Medium supplemented with Vascular Smooth Muscle Growth Kit. The Human Umbilical Vein Endothelial Cells (HUVEC) were purchased from Lonza (#CC-2935) and cultured using commercial media as per the manufacturers’ instructions. HASMC were further immortalized using hTERT lentivirus infection to extend the life span and achieve a higher transfection efficiency (1, 2). Cells were incubated in 95% O2 and 5% CO2 at 37°C for regular maintenance.

Antibodies for β-actin (sc-47778), eNOS (sc-376751), GLUT4 (sc-53566) and NFκB p65 (sc-8008) were obtained from Santa Cruz Biotechnology (Shanghai, China). The antibodies for CD31 (ab24590), Histone H3 lysine 9 dimethylation (H3K9me2, #ab1220), Histone H3 lysine 9 trimethylation (H3K9me3, #ab8898), H3 lysine 27 dimethylation (H3K27me2, #ab24684) and H3 lysine 27 trimethylation (H3K27me3, #ab6002), H2AX (ab20669), Phospho-Ser139 Histone H2A.X (γH2AX, #ab2893) and Nrf2 (#ab137550) were obtained from Abcam. The transcriptional activity of NFκB p65 was determined by the NFκB p65 Transcription Factor Assay Kit (#ab133112 from Abcam) according to manufacturers’ instructions. Nuclear extracts were prepared using the NE-PER Nuclear and Cytoplasmic Extraction Reagents Kit (#78833, Pierce Biotechnology). Protein concentration was measured using the Coomassie Protein Assay Kit (#23200, Pierce Biotechnology). Betulinic acid (BA, #B8936) and streptozotocin (STZ, #S0130) were obtained from Sigma (Shanghai, China). BA was dissolved in DMSO (dimethyl sulfoxide) to make a stock solution, and the final concentration of the above solvents did not exceed 0.1% in any experiment.

**Construction of GLUT4 reporter plasmid.** Human genomic DNA was prepared from HASMC cells. In order to construct the GLUT4 reporter plasmid, the gene promoter (2kb upstream of the transcription start site plus first exon) was amplified from Ensembl gene ID: TRARG1-201 ENST00000333813.4 (for GLUT4) by PCR and subcloned into the pGL3-basic vector (# E1751, Promega) using underlined restriction sites with the following primers: GLUT4 forward: 5’-gcgc-acgcgt- cca agg tct tca ttt tgc aaa -3’ (Mlu I) and GLUT4 reverse: 5’- gtac- aagctt-cat gat gga aat gat gag ggg -3’ (Hind III). To map GLUT4 promoter activity, the related deletion promoter constructs were generated by PCR methods and subcloned into the pGL3-basic vector. All the vectors were verified by sequencing, and detailed information on these plasmids is available upon request (3).

**Luciferase reporter assay.** 1.0×10^5^ of treated cells were seeded in a 6-well plate with complete medium to grow until they reached 80% confluence. Cells were then cotransfected by 3µg of full length or deletion reporter constructs, together with 0.2µg of pRL-CMV-Luc *Renilla* plasmid (from Promega). After treatment, the cells were harvested and the luciferase activity assays were carried out using the Dual-Luciferase^TM^ Assay System (Promega) The transfection efficiencies were normalized using a cotransfected *Renilla* plasmid, and the reporter activity was calculated according to manufacturers’ instructions (3).

**DNA methylation analysis.** We developed a real-time PCR-based method for methylation-specific PCR (MSP) analysis on the human GLUT4 promoter according to the previously described method with some modifications (4-6). Genomic DNA from human HASMC was extracted and purified before then being treated by bisulfite modification using the EpiJET Bisulfite Conversion Kit (#K1461, Fisher). The modified DNA was then amplified using methylated and unmethylated primers for MSP that were designed using the Methprimer software: (<http://www.urogene.org/cgi-bin/methprimer/methprimer.cgi>) with the below details: Methylated primer: forward 5’- gtt ttt tcg agt tgg tat ttg ttc -3’, reverse 5’- aac ccc ata aat aaa ttt cta cgt a -3’; Unmethylated primer: forward 5’- ttt ttt gag ttg gta ttt gtt tgg -3’; reverse 5’- aac ccc ata aat aaa ttt cta cat a -3’. Product size: 166bp (methylated) & 164bp (unmethylated); CpG island size: 231bp; Tm: 63.8°C. The final methylation readout was normalized by unmethylated input PCR (7).

**RT reaction and real-time quantitative PCR**. Total RNA from treated cells was extracted using the RNeasy Micro Kit (Qiagen) and the RNA was reverse transcribed using an Omniscript RT kit (Qiagen). All the primers were designed using Primer 3 Plus software with the Tm at 60°C, primer size of 21bp, and the product length in the range of 140-160bp (see Table S1). The primers were validated with an amplification efficiency in the range of 1.9-2.1 and the amplified products were confirmed with agarose gel. The real-time quantitative PCR was run on iCycler iQ (Bio-Rad) with the Quantitect SYBR green PCR kit (Qiagen). The PCR was performed by denaturing at 95°C for 8 min followed by 45 cycles of denaturation at 95°C, annealing at 60°C, and extension at 72°C for 10s, respectively. 1 µl of each cDNA was used to measure target genes. β-actin was used as the housekeeping gene for transcript normalization, and the mean values were used to calculate relative transcript levels with the ^ΔΔ^CT method per instructions from Qiagen. In brief, the amplified transcripts were quantified by the comparative threshold cycle method using β-actin as a normalizer. Fold changes in gene mRNA expression were calculated as 2^−ΔΔCT^ with CT = threshold cycle, ΔCT=CT (target gene)-CT(β-actin), and the ΔΔCT =ΔCT (experimental)-ΔCT (reference) (3, 7).

**Western Blotting.** The cells were lysed in an ice-cold lysis buffer (0.137M NaCl, 2mM EDTA, 10% glycerol, 1% NP-40, 20mM Tris base, pH 8.0) with protease inhibitor cocktail (Sigma). The proteins were separated in 10% SDS-PAGE and further transferred to the PVDF membrane. The membrane was incubated with appropriate antibodies, washed and incubated with HRP-labeled secondary antibodies, and then the blots were visualized using the ECL+plus Western Blotting Detection System (Amersham). The blots were quantitated by IMAGEQUANT, and the results were normalized by β-actin (3).

**Chromatin Immunoprecipitation (ChIP).** Cells were washed and crosslinked using 1% formaldehyde for 20 min and terminated by 0.1M glycine. Cell lysates were sonicated and centrifuged. 500 µg of protein were pre-cleared by BSA/salmon sperm DNA with preimmune IgG and a slurry of Protein A Agarose beads. Immunoprecipitations were performed with the indicated antibodies, BSA/salmon sperm DNA and a 50% slurry of Protein A agarose beads. Input and immunoprecipitates were washed and eluted, then incubated with 0.2mg/ml Proteinase K for 2h at 42˚C, followed by 6h at 65˚C to reverse the formaldehyde crosslinking. DNA fragments were recovered by phenol/chloroform extraction and ethanol precipitation. A ~150 bp fragment on the promoter was amplified by real-time PCR (qPCR) using the primers provided in Table S1 (3).

**In vivo rat experiments**. The animal protocol conformed to US NIH guidelines (Guide for the Care and Use of Laboratory Animals, No. 85-23, revised 1996), and was reviewed and approved by the Institutional Animal Care and Use Committee. The male Wistar rats were housed 4 or 5 per cage on a 12:12-h light-dark cycle and given commercial rodent chow and water ad libitum on arrival.

Rat Protocol 1: Generation of diabetic rats. 2-month-old rats were fed by a high-fat diet for one month followed by injection of 35 mg/kg streptozotocin (STZ, 0.05 M sodium citrate, pH 5.5) after an 8-hour fasting period. Blood glucose levels were monitored continuously for 4 weeks after injection and the animals with blood glucose >11.1mM were considered to be diabetes positive. Control (CTL) rats received only vehicle injection (8, 9).

Rat Protocol 2: Development of cutaneous burns in rats. The diabetic rats from Protocol 1 described above were subjected to a model of cutaneous burn injury after 2 weeks of STZ injection. The dorsum of each rat was shaved with electric clippers and depilated with Nair. The rats were anesthetized by inhalation of 5% isoflurane and then the cutaneous burn injury was made on the dorsa of the rats by exposure to a hot copper pillar (2-cm diameter) at 75ºC for 15 seconds. The subsequent wound healing process was then monitored and evaluated (8, 9).

Rat Protocol 3: BA treatments of cutaneous burns in diabetic rat model. The above rats from Protocol 2 received treatments of either vehicle (VEH) or BA administration. For BA treatment, the BA was first dissolved in 1% DMSO, then diluted 10 times in 0.9 % NaCl solution, and administered intraperitoneally every 3 days at a dose of 10 mg/(kg body weight) for 4 weeks starting from one week before the burn injury. On the other hand, the dissolved BA was administrated topically by spraying in a dose of 20 μM each day continuously for 3 weeks starting from the second day after the introduction of burn injury. The experimental rats were randomly separated into 4 groups as follows: Group 1: Control (CTL) rats that received vehicle (VEH) treatment (CTL/VEH); Group 2: STZ-induced diabetic (STZ) rats that received VEH treatment (STZ/VEH); Group 3: STZ-induced diabetic (STZ) rats that received BA topical administration treatment (STZ/BA-TOP); Group 4: STZ-induced diabetic (STZ) rats that received BA intraperitoneal administration treatment (STZ/BA-IP); Group 5: STZ-induced diabetic (STZ) rats that received BA administration both topically and intraperitoneally (STZ/BA-IP/TOP). The wound healing process was monitored and evaluated throughout the treatment. After treatment, the animals were subjected to glucose/insulin tolerance tests. The whole blood was then withdrawn by heart puncture and the serum was prepared by centrifugation. Peripheral blood mononuclear cells (PBMC) were isolated from the blood using Ficoll-Paque Plus lymphocyte separation medium, the fibroblast cells were isolated from underarm area of skin from treated mice for in vitro biological assays (10). Rats were sacrificed and the wound tissues and soleus muscles were collected.

Rat Protocol 4: Wound healing measurement. Digital photographs of the wounds were taken every two days for 21 days. Wound area was quantified as a percentage area of the original wound size using Image J software. At indicated time points, wounds were excised and snap-frozen or, alternatively, processed for either H&E staining or immunohistochemistry (IHC). Images were taken using a Carl Zeiss MIRAX MIDI slide scanner, and the analyses were performed using a 3DHISTECH Pannoramic Viewer for the quantification of granulation tissue deposition (11). Vascular density was detected on frozen sections by IHC using CD31 mouse monoclonal antibody. For quantification of CD31 positivity, wounds were analyzed under 200ˣ magnification, and the number of positive 6 cells per high-power field (HPF) were counted. All counts and observations were performed by a blinded observer (12). Cytokine levels from rat serum were measured using ELISA kits from R&D Systems and the peritoneal macrophage was isolated for gene expression (8, 9, 12).

**Analysis of glucose uptake**. Treated cells and the soleus muscles were used for this assay. The soleus muscles were isolated from treated rats and were dissected, weighed, pre-incubated (30 min) and then incubated (60 min) at 37ºC in Krebs Ringer-bicarbonate (KRb) buffer with a composition of 122mM NaCl, 3mM KCl, 1.2mM MgSO4, 1.3mM CaCl2, 0.4mM KH2PO4, and 25mM NaHCO3 and bubbled with O2/CO2 (95%:5%, v/v) until pH 7.4. The [U-^14^C]-2-deoxy-D-glucose (^14^C-DG) (0.1μCi/mL) was added to each sample during the incubation. After incubation, the muscles were placed in screw cap tubes containing 1 mL of distilled water and frozen at −20ºC in a freezer followed by 10 min of boiling. 25 μL aliquots of tissue and external medium were placed in a scintillation counter for the radioactivity measurements (13), protein concentration was measured using the Coomassie Protein Assay Kit (Pierce Biotechnology), and the glucose uptake results were expressed as counts per minute (CPM)/ml incubation medium (14).

**Intraperitoneal glucose tolerance test (IPGTT).** For the glucose tolerance test, pre-treated mice were administrated by intraperitoneal injection of glucose (2g/kg body weight) after a 6-hour fasting period. The blood samples were collected from the tail vein and blood glucose was monitored using a OneTouch Ultra^®^2 Glucometer at the time points of 0, 15, 30, 60, 90 and 120 min after injection. The serum insulin levels were evaluated by Rat Insulin ELISA Kit (#ERINS from Invitrogen) according to manufacturers’ instructions (15, 16).

**Isolation of rat PBMC cells**. The heparinized peripheral blood was collected from rats by puncturing the heart and was diluted 1:3 with Hank's balanced salts solution without Ca^2+^/Mg^2+^ (HBSS solution). The diluted blood was layered onto 15ml of Ficoll-Paque in 50 ml sterile centrifuge tubes followed by centrifugation at 300×g at 20ºC for 40 min. The PBMC layers were then harvested and washed three times with HBSS solution. The pellets were then resuspended with lysing buffer containing 150mM NH4Cl, 1.0mM KHCO3, and 0.1mM Na2EDTA, pH 7.4 and incubated for 5 min at room temperature to remove contaminated red cells. The cell suspensions were then centrifuged and washed twice with HBSS solution, and then the cell pellet was resuspended for further biological assays.

**Measurement of oxidative stress.** Treated cells were seeded in a 24-well plate and incubated with 10 μM CM-H2DCFDA (Invitrogen) for 45 min at 37°C. The intracellular formation of reactive oxygen species (ROS) was then measured at excitation/emission wavelengths of 485/530nm using a FLx800 microplate fluorescence reader (Bio-Tek) and the data was normalized as arbitrary units (3, 17). The GSH/GSSG ratio was determined by GSH/GSSG Ratio Detection Assay Kit (Fluorometric - Green) (#ab205811 from Abcam), the transcriptional activity of Nrf2 was determined by Nrf2 Transcription Factor Assay Kit (Colorimetric) (#ab207223 from Abcam), the Methylglyoxal (MG) generation was evaluated using Methylglyoxal Assay Kit (#ab241006 from Abcam) and the 3-nitrotyrosine (3-NT) was measured using 3-Nitrotyrosine ELISA Kit (#ab116691 from Abcam) according to manufacturers’ instructions.

Measurement of DNA breaks. The 8-hydroxy-2'-deoxyguanosine (8-OHdG) formation was measured using an OxiSelect™ Oxidative DNA Damage ELISA Kit (Cat No. STA320, from Cell Biolabs Inc.) according to manufacturers’ instructions. The formation of γH2AX was measured from nuclear extracts by western blotting using H2AX as the input control.

**Immunostaining**. The treated cells were transferred to cover slips and the cells were fixed in 4% paraformaldehyde for 20 min before being incubated with 0.3% Triton X-100 in PBS for 15 min. After blocking with 5% goat serum in PBS at room temperature for 30 min, cells were incubated with 8-oxo-dG anti-mouse antibody (# 4354-MC-050, from Novus Biologicals) for 12 h at 4°C and subsequently with secondary antibody Alexa Fluor 488. The cover slips were then mounted by antifade Mountant with DAPI (staining nuclei, in blue). The photographs were taken using a [Confocal Laser Microscope](https://www.sogou.com/link?url=DSOYnZeCC_qw-OVKG_MsR3KENashJ6PPMhOejy_Q5JJflCntg_rzjU2lo9-QKkufX5Qp7YP6841C08P_Gzn4lQD4cR4JDdkk5sef3Ee0PfoOX3hBKf-DUA..) (Leica, 20x lens) and quantitated by Image J. software (18).

# **Cytokine analysis by ELISA.** Rat interleukins from either supernatant or serum, including IL1β, IL6 and MCP1, were measured using the Rat IL-1β/IL-1F2 Quantikine ELISA Kit (#RLB00), Rat IL-6 Quantikine ELISA Kit (#RRA00), and Rat JE/MCP-1/CCL2 DuoSet ELISA Kit (#DY3144-05), respectively, according to manufacturer’s instructions from R&D Systems (19).

**Immunohistochemistry.** The tissues were dissected and snap-frozen in the OCT compound. The 10 μm sections were cut by clean microtome, mounted on PEN-membrane slides (2.0μm, Leica), and stored at -20ºC before use. The slides were first fixed by 3.7% formaldehyde at 37ºC for 15 min, permeabilized by 1% BSA+0.2% Triton X-100 in PBS for 1 hour, and then blotted with 40 μg/ml (dilute 1:20) of either 8-oxo-dG, 3-Nitrotyrosine, or CD31 mouse monoclonal antibody for 2 hours. They were then washed three times and the 3,3'-diaminobenzidine (DAB) labeled anti-mouse secondary antibody (1:200) was added for blotting for another 1 hour. After thorough washing, the slides were visualized and photographed. The relative densities of each group were quantitated for protein expression using Image J. software (2, 8, 9).

**In vivo superoxide anion (O_2_^.-^) release**. Superoxide anion release in amygdala tissues were extracted by dimethyl sulfoxide-tetrabutylammonium chloride (DMSO-TBAC) solution and the TBAC-O_2_^.-^ complex was then further detected by use of the luminol-EDTA-Fe enhanced chemiluminescence system (20). Briefly, the biological tissues were isolated and purged continuously by N2 gas to remove traces of oxygen, and O_2_^.-^ from tissues was extracted by DMSO-TBAC, then the chemiluminescent reagents and O_2_^.-^ extract solutions were pumped into a glass scintillation vial which was placed in the luminometer, and the chemiluminescence intensity was continuously monitored for 2min and calculated. Superoxide levels were calculated from the standard curve generated by the xanthine/xanthine oxidase reaction (17).

**Statistical analysis**. The data was given as mean ± SD; all of the experiments were performed at least in quadruplicate unless otherwise indicated. The one-way ANOVA followed by the Bonferroni post hoc test was used to determine statistical significance of different groups under normal distribution. SPSS 22 software was used for statistical analysis and a *P* value < 0.05 was considered significant (8, 9).

REFERENCES

1. Bodnar AG, Ouellette M, Frolkis M, Holt SE, Chiu CP, Morin GB, et al. Extension of life-span by introduction of telomerase into normal human cells. *Science.* 1998;279(5349):349-52.

2. Li H, Liu Z, Gou Y, Yu H, Siminelakis S, Wang S, et al. Estradiol mediates vasculoprotection via ERRalpha-dependent regulation of lipid and ROS metabolism in the endothelium. *J Mol Cell Cardiol.* 2015;87:92-101.

3. Zhang H, Li L, Li M, Huang X, Xie W, Xiang W, et al. Combination of betulinic acid and chidamide inhibits acute myeloid leukemia by suppression of the HIF1alpha pathway and generation of reactive oxygen species. *Oncotarget.* 2017;8(55):94743-58.

4. Ogino S, Kawasaki T, Brahmandam M, Cantor M, Kirkner GJ, Spiegelman D, et al. Precision and performance characteristics of bisulfite conversion and real-time PCR (MethyLight) for quantitative DNA methylation analysis. *J Mol Diagn.* 2006;8(2):209-17.

5. Eads CA, Danenberg KD, Kawakami K, Saltz LB, Blake C, Shibata D, et al. MethyLight: a high-throughput assay to measure DNA methylation. *Nucleic Acids Res.* 2000;28(8):E32.

6. Nosho K, Irahara N, Shima K, Kure S, Kirkner GJ, Schernhammer ES, et al. Comprehensive biostatistical analysis of CpG island methylator phenotype in colorectal cancer using a large population-based sample. *PLoS ONE.* 2008;3(11):e3698.

7. Zou Y, Lu Q, Zheng D, Chu Z, Liu Z, Chen H, et al. Prenatal levonorgestrel exposure induces autism-like behavior in offspring through ERβ suppression in the amygdala. *Mol Autism.* 2017;8:46.

8. Li M, Yu H, Pan H, Zhou X, Ruan Q, Kong D, et al. Nrf2 Suppression Delays Diabetic Wound Healing Through Sustained Oxidative Stress and Inflammation. *Front Pharmacol.* 2019;10:1099.

9. Zhou X, Li M, Xiao M, Ruan Q, Chu Z, Ye Z, et al. ERβ Accelerates Diabetic Wound Healing by Ameliorating Hyperglycemia-Induced Persistent Oxidative Stress. *Front Endocrinol (Lausanne).* 2019;10:499.

10. Seluanov A, Vaidya A, and Gorbunova V. Establishing primary adult fibroblast cultures from rodents. *J Vis Exp.* 2010(44).

11. Zhang H, Li L, Chen Q, Li M, Feng J, Sun Y, et al. PGC1beta regulates multiple myeloma tumor growth through LDHA-mediated glycolytic metabolism. *Mol Oncol.* 2018;12(9):1579-95.

12. Thangarajah H, Yao D, Chang EI, Shi Y, Jazayeri L, Vial IN, et al. The molecular basis for impaired hypoxia-induced VEGF expression in diabetic tissues. *Proc Natl Acad Sci U S A.* 2009;106(32):13505-10.

13. Cazarolli LH, Folador P, Moresco HH, Brighente IM, Pizzolatti MG, and Silva FR. Mechanism of action of the stimulatory effect of apigenin-6-C-(2''-O-alpha-l-rhamnopyranosyl)-beta-L-fucopyranoside on 14C-glucose uptake. *Chem Biol Interact.* 2009;179(2-3):407-12.

14. Castro AJ, Frederico MJ, Cazarolli LH, Bretanha LC, Tavares Lde C, Buss Zda S, et al. Betulinic acid and 1,25(OH)(2) vitamin D(3) share intracellular signal transduction in glucose homeostasis in soleus muscle. *Int J Biochem Cell Biol.* 2014;48:18-27.

15. Song TJ, Park CH, In KR, Kim JB, Kim JH, Kim M, et al. Antidiabetic effects of betulinic acid mediated by the activation of the AMP-activated protein kinase pathway. *PLoS One.* 2021;16(4):e0249109.

16. Tahara A, Matsuyama-Yokono A, Nakano R, Someya Y, and Shibasaki M. Effects of antidiabetic drugs on glucose tolerance in streptozotocin-nicotinamide-induced mildly diabetic and streptozotocin-induced severely diabetic mice. *Horm Metab Res.* 2008;40(12):880-6.

17. Yao D, Shi W, Gou Y, Zhou X, Yee Aw T, Zhou Y, et al. Fatty acid-mediated intracellular iron translocation: a synergistic mechanism of oxidative injury. *Free Radic Biol Med.* 2005;39(10):1385-98.

18. Wang X, Lu J, Xie W, Lu X, Liang Y, Li M, et al. Maternal diabetes induces autism-like behavior by hyperglycemia-mediated persistent oxidative stress and suppression of superoxide dismutase 2. *Proc Natl Acad Sci U S A.* 2019;116(47):23743-52.

19. Kobayashi EH, Suzuki T, Funayama R, Nagashima T, Hayashi M, Sekine H, et al. Nrf2 suppresses macrophage inflammatory response by blocking proinflammatory cytokine transcription. *Nat Commun.* 2016;7:11624.

20. Yao D, Vlessidis AG, Gou Y, Zhou X, Zhou Y, and Evmiridis NP. Chemiluminescence detection of superoxide anion release and superoxide dismutase activity: modulation effect of Pulsatilla chinensis. *Anal Bioanal Chem.* 2004;379(1):171-7.

**Table S1. Sequences of primers for the real time quantitative PCR (qPCR)**

| Gene | Species | Analysis | Forward primer (5'→3') | Reverse primer (5'→3') |
| --- | --- | --- | --- | --- |
| β-actin | Human | mRNA | gatgcagaaggagatcactgc | atactcctgcttgctgatcca |
| eNOS | Human | mRNA | cagctagccaaagtcaccatc | tgatggaaaacaggagtgagg |
| GLUT4 | Human | mRNA | attggcatgggtttccagtat | gcagctgagatctggtcaaac |
| Nrf2 | Human | mRNA | agatagtgcccctggaagtgt | caggcaattctttctctggtg |
| NFκB p65 | Human | mRNA | atcccatctttgacaatcgtg | gtcccgtgaaatacacctcaa |
| GLUT4 | Human | ChIP | ctggagctgcaacagaaacc | ttccttttcaattcccctctc |
| β-actin | Rat | mRNA | ttccttcctgggtatggaatc | cttctgcatcctgtcagcaat |
| eNOS | Rat | mRNA | gcatcacctacgataccctca | ttgacccaatagctgctcagt |
| Nrf2 | Rat | mRNA | cagcttttggcagagacattc | taaatcagtcatggccgtctc |
| IL1β | Rat | mRNA | gagagtgtggatcccaaacaa | ggaagacaggtctgtgctctg |
| IL6 | Rat | mRNA | agccagagtcattcagagcaa | gtcttggtccttagccactcc |
| MCP1 | Rat | mRNA | tcacctgctgctactcattca | attccttattggggtcagcac |
| NFκB p65 | Rat | mRNA | ctcgcatccgatttttgataa | gtcccgtgaaatacacctcaa |
| GLUT4 | Rat | mRNA | aaggtggcaggaagaagtctc | ccaaaaagtcatccttgtcca |

FIGURE S1

**Figure S1. Representative pictures of full blots for Western Blotting.** (a) Representative full blots for Figure 1c. (b) Representative full blots for Figure 1d. (c). Representative full blots for Figure 2c. (d). Representative full blots for Figure 4c. (e). Representative full blots for Figure 5c. (f). Representative full blots for Figure 5d.

FIGURE S2

**Figure S2.** **Potential effect of BA and hyperglycemia on epigenetic modifications on the GLUT4 promoter.** HASMC were treated by either low glucose (LG, 5mM), high glucose (HG, 25mM), or HG plus 20uM BA for 4 days, and the cells were harvested for ChIP analysis. (a) Histone H4 methylation on the GLUT4 promoter, n=4. (b) Histone acetylation on the GLUT4 promoter using H3K9,14,18,23,27ac and H4K5,8,12,16ac antibodies, n=4. Data were expressed as mean ± SD.

FIGURE S3

**Figure 3. BA reverses diabetes-mediated GLUT4 suppression by modulation of histone methylation on the GLUT4 promoter in fibroblasts**. Fibroblast cells were isolated from skin of treated animal from either control (CTL) or STZ-mediated diabetic (STZ) group, then the cells were treated by either vehicle (VEH) or 20µM of BA for 24 hours, and the cells were harvested for biological assays. (a) mRNA levels by qPCR, n=4. (b) ChIP analysis on GLUT4 promoter. n=4. (c) ^14^C-DG uptake assay, n=5. *, *P*<0.05, vs CTL/VEH group; ¶, *P*<0.05, vs STZ/VEH group. Data were expressed as mean ± SD.
